# An In Silico and an In Vitro Inhibition Analysis of Glycogen Phosphorylase by Flavonoids, Styrylchromones, and Pyrazoles

**DOI:** 10.3390/nu14020306

**Published:** 2022-01-12

**Authors:** Sónia Rocha, Natália Aniceto, Rita C. Guedes, Hélio M. T. Albuquerque, Vera L. M. Silva, Artur M. S. Silva, Maria Luísa Corvo, Eduarda Fernandes, Marisa Freitas

**Affiliations:** 1 LAQV-REQUIMTE, Laboratory of Applied Chemistry, Department of Chemical Sciences, Faculty of Pharmacy, University of Porto, 4050-313 Porto, Portugal; up201607090@edu.ff.up.pt (S.R.); egracas@ff.up.pt (E.F.); 2 Research Institute for Medicines, Faculdade de Farmácia, Universidade de Lisboa, 1649-003 Lisboa, Portugal; nataliaaniceto@ff.ul.pt (N.A.); ritaguedes@campus.ul.pt (R.C.G.); lcorvo@ff.ulisboa.pt (M.L.C.); 3 LAQV-REQUIMTE, Department of Chemistry, University of Aveiro, 3810-193 Aveiro, Portugal; helio.albuquerque@ua.pt (H.M.T.A.); verasilva@ua.pt (V.L.M.S.); artur.silva@ua.pt (A.M.S.S.)

**Keywords:** natural compounds, synthetic compounds, glycogen phosphorylase, structure–activity relationship, type 2 diabetes

## Abstract

Glycogen phosphorylase (GP) is a key enzyme in the glycogenolysis pathway. GP inhibitors are currently under investigation as a new liver-targeted approach to managing type 2 diabetes *mellitus* (DM). The aim of the present study was to evaluate the inhibitory activity of a panel of 52 structurally related chromone derivatives; namely, flavonoids, 2-styrylchromones, 2-styrylchromone-related derivatives [2-(4-arylbuta-1,3-dien-1-yl)chromones], and 4- and 5-styrylpyrazoles against GP, using in silico and in vitro microanalysis screening systems. Several of the tested compounds showed a potent inhibitory effect. The structure–activity relationship study indicated that for 2-styrylchromones and 2-styrylchromone-related derivatives, the hydroxylations at the A and B rings, and in the flavonoid family, as well as the hydroxylation of the A ring, were determinants for the inhibitory activity. To support the in vitro experimental findings, molecular docking studies were performed, revealing clear hydrogen bonding patterns that favored the inhibitory effects of flavonoids, 2-styrylchromones, and 2-styrylchromone-related derivatives. Interestingly, the potency of the most active compounds increased almost four-fold when the concentration of glucose increased, presenting an IC_50_ < 10 µM. This effect may reduce the risk of hypoglycemia, a commonly reported side effect of antidiabetic agents. This work contributes with important considerations and provides a better understanding of potential scaffolds for the study of novel GP inhibitors.

## 1. Introduction

Diabetes *mellitus* (DM) is a global chronic metabolic disorder and is one of the leading causes of mortality and a reduced life expectancy [1]. Overall, DM significantly increases the incidence of other chronic health problems, such as cardiovascular diseases, retinopathy, neuropathy, and nephropathy [2]. Type 2 DM is the most common form of DM and is mainly caused by insulin resistance in insulin-sensitive tissues, with a progressive decline of β-cell function. These abnormalities result in chronic elevations in glycemia, known as hyperglycemia [3,4,5].

The rising prevalence of type 2 DM has created an urgent impetus for the development of new approaches for glycemic control in diabetic patients, in order to maintain plasma glucose concentrations in the normal range, avoiding the development of complications [4]. Among the several options of antidiabetic drugs, metformin is the most widely prescribed drug across all age groups. Metformin acts by decreasing hepatic gluconeogenesis and by increasing the hepatic uptake of glucose [6]. Despite being the most widely prescribed antidiabetic drug, it has been associated with gastrointestinal side effects. Therefore, there is an urgent need for effective medical management for type 2 DM [7,8].

As a key player in the maintenance of glucose homeostasis, accounting for nearly 90% of endogenous glucose production, the liver is an attractive therapeutic target. Additionally, insulin resistance, which is the main feature of type 2 DM, was shown to be correlated with excessive hepatic glucose production [4]. Liver-targeted approaches to control glucose homeostasis are currently under investigation. However, new effective therapeutic agents have not been approved [7,9]. Some novel therapeutic strategies to target hepatic dysregulation include the specific metabolic enzymes of gluconeogenesis and glycogenolysis. A novel liver-targeted approach for the management of type 2 DM includes glycogen phosphorylase (GP, EC 2.4.1.1) inhibitors [7].

**Figure 1:**
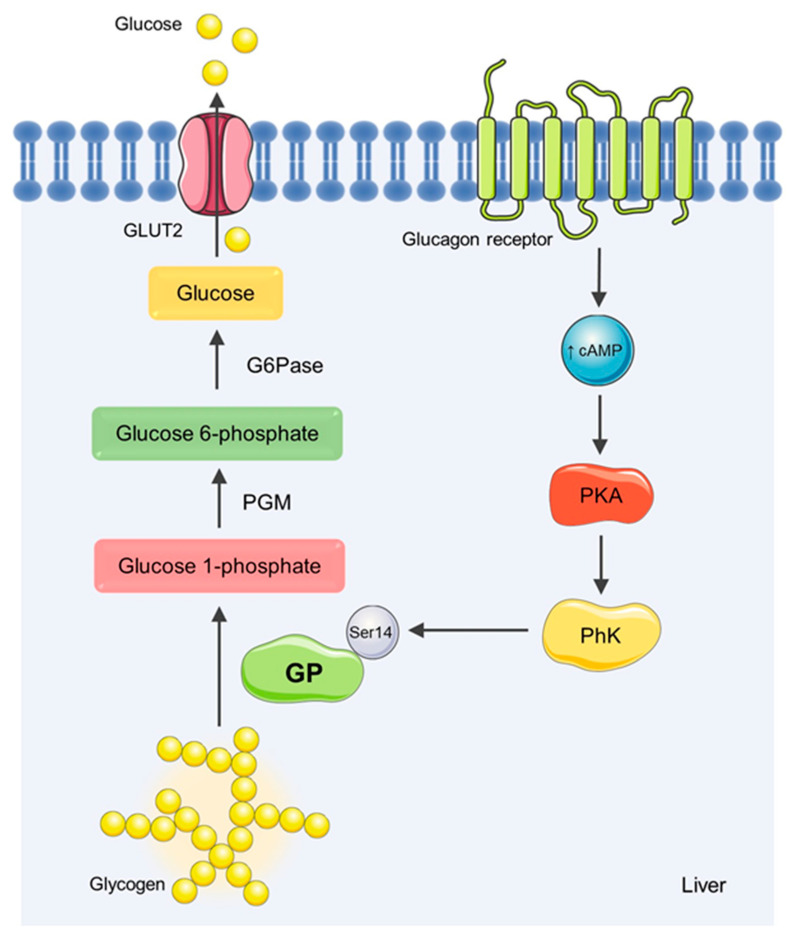
Regulation of hepatic glycogenolysis. Under fasting conditions, glycogenolysis is activated and glycogen synthesis is suppressed. The activation of glucagon receptor induces the increase in intracellular cyclic AMP (cAMP) and the consequent activation of protein kinase A (PKA). PKA is responsible for the phosphorylation of phosphorylase kinase (PhK), which, in turn, activates GP by serine-14 phosphorylation. GP, as a key enzyme involved in glycogenolysis, catalyzes the removal of a glucose residue of the glycogen chain, generating glucose 1-phosphate. Glucose 1-phosphate is converted to glucose 6-phosphate by phosphoglucomutase (PGM), and glucose 6-phosphatase (G6Pase) converts glucose 6-phosphate into glucose, which can be transported to the bloodstream. This process is mediated by glucose transporter 2 (GLUT2). Image bank [17].

GP is a key enzyme in the glycogenolysis pathway (Figure 1), with crucial roles in the hepatic glycogen content and glucose homeostasis. Under physiologic conditions, this enzyme catalyzes the degradation of glycogen, at the α-1,4-glycosidic linkage, to produce glucose 1-phosphate monomers in the presence of inorganic phosphate [10,11]. GP exists as a dimer, with two equal subunits of about 97 kDa [12]. The enzyme can be found in two different states, since phosphorylation of serine-14 by phosphorylase kinase (PhK) converts the inactive dephosphorylated form, GPb, into an active phosphorylated form, GPa [10]. Three isoforms have been identified, which are located at different metabolically active tissues, with different physiological functions, namely the brain (bGP), the liver (lGP), and muscle tissue (mGP). The lGP isoform ensures that the body’s glycemic demands are received from hepatic glycogen stores. As the key isoform involved in glucose homeostasis, lGP is the focus of therapeutic interventions for the management of type 2 DM [13,14]. Therefore, the pharmacological inhibition of lGP may reduce plasma glucose levels by increasing glucose storage in the form of glycogen, limiting the pathogenic consequences related to hyperglycemia [15]. Although GP inhibitors have been considered as a promising therapeutic approach, these inhibitors have not yet reached the clinic. One of the major barriers has been the isoform specificity [14]. The mammalian brain, liver, and skeletal muscle isoforms of GP share nearly 80% of their sequence, and almost 100% of homology in the catalytic site. Hence, finding inhibitors able to selectively bind lGP remains a difficult task [16]. The inhibition of skeletal mGP could lead to serious adverse effects in muscle functions, such as muscle cramping, which is related to the accumulation of glycogen in the tissue. Thus, the design of selective and effective inhibitors towards lGP remains a crucial topic of research [12,14].

**Figure 2:**
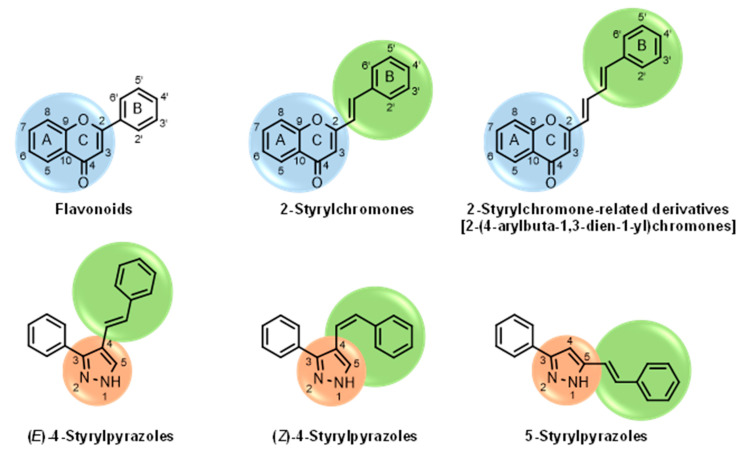
General chemical structures and numerical representations of the tested chromone derivatives, flavonoids, 2-styrylchromones, and 2-styrylchromone-related derivatives [2-(4-arylbuta-1,3-dien-1-yl)chromones], and 4- and 5-styrylpyrazoles. The chromone core is represented in blue, the styryl group is represented in green, and the pyrazole core in orange.

Several natural and synthetic compounds have been reported to inhibit GP [10,13,16,18]. However, the identification of GP inhibitors with new scaffolds needs to be explored. As a privileged structure in drug discovery, chromone (4*H*-chromen-4-one) is a promising template for new compounds. Chromones are a family of naturally occurring compounds ubiquitous in nature. The diversity of this family of compounds has attracted many researchers due to their recognized pharmacological properties, particularly their anti-inflammatory, antimicrobial, and antidiabetic activities [19,20,21]. Flavonoids, are the most abundant polyphenols in the human diet, holding a chromone core. This is a class of plant secondary metabolites widely distributed in nature and are well recognized by their ability to modulate a large number of DM targets [22,23,24,25]. 2-Styrylchromones and 2-styrylchromone-related derivatives [2-(4-arylbuta-1,3-dien-1-yl)chromones], which are structurally related to flavonoids, also belong to the chromone family, and hold a styryl and a 4-arylbuta-1,3-dien-1-yl moiety, respectively, at C-2 of the chromone core. The presence of 2-styrylchromones in nature is scarce, and their origin is mostly synthetic, while the 2-styrylchromone-related derivatives are only synthetic compounds. Nonetheless, a large variety of biological activities have been reported for these compounds [26,27]. Another family of promising heterocyclic compounds are pyrazoles. These five-membered heterocycles possess a promising framework found in diverse commercially available medicines, such as anti-inflammatory and anti-cancer formulations [28]. Hence, the aim of the present work is to study the inhibitory activities of a panel of 52 structurally related chromone derivatives (flavonoids, 2-styrylchromones, and 2-styrylchromone-related derivatives) and 4- and 5-styrylpyrazoles (Figure 2). Most of these compounds were assessed here for the first time. The activities of some selected compounds were evaluated in the absence and presence of high glucose levels, to mimic hyperglycemic conditions. Additionally, molecular docking calculations were also performed to complement the in vitro enzymatic studies.

## 2. Materials and Methods

### 2.1. Chemicals

The following reagents were obtained from Sigma-Aldrich, Inc. (St. Louis, MO): dimethylsulfoxide (DMSO), 5-chloro-*N*-[(1*S*,2*R*)-3-(dimethylamino)-2-hydroxy-3-oxo-1-(phenylmethyl)propyl]-1*H*-indole-2-carboxamide (**CP-91149**), HEPES, MgCl_2_, glucose 1-phosphate, glycogen, and compounds **42**, **45** and **47**. KCl was obtained from José M. Vaz Pereira, S.A. (Sintra, Lisboa). The rabbit muscle phosphorylated form, mGPa, was obtained from Creative Enzymes^®^ (Shirley, NY, USA). The reagent for colorimetric phosphate quantitation, BIOMOL^®^ Green, was obtained from Enzo Life Sciences, Inc. (Postfach, Lausen, Switzerland). Compounds **36**–**41** and **44** were obtained from INDOFINE Chemical Company, Inc. (Hillsborough, NJ, USA). Compounds **46** and **48**–**52** were obtained from Extrasynthese (Lyon Nord, Genay, France). Compound **43** was obtained from Alfa Aesar (Erlenbachweg, Kandel, Germany). Compounds **1**–**35** were synthesized as previously described [27,29,30,31,32,33,34,35].

### 2.2. The In Vitro Glycogen Phosphorylase Inhibition Assay

The GP activity was measured using a previously optimized and validated method [36]. In each assay, GP activity was measured by the direction of glycogen synthesis, which involved monitoring the colorimetric determination of inorganic phosphate deriving from glucose 1-phosphate. Briefly, in a 96-well plate, the rabbit mGPa (0.38 U/mL), dissolved in a 50 mM HEPES buffer solution with a pH of 7.2, was exposed to the compounds (0–50 µM) under study that were dissolved in DMSO for 15 min at 37 °C. The enzymatic reaction was then started with the addition of a 50 mM HEPES buffer solution, with a pH of 7.2, containing 100 mM KCl, 2.5 mM MgCl_2_, 0.25 mM glucose 1-phosphate, and 0.25 mg/mL glycogen incubated for 30 min at 37 °C. After this incubation time, the reagent for colorimetric phosphate quantitation, BIOMOL^®^ Green, was added to the reaction mixture. The reaction was monitored in a microplate reader (Synergy HT, BioTek Instruments, Winooski, VT, USA) at 620 nm. The results of the in vitro inhibitory activity of GPa corresponded to the absorbance value at 15 min, expressed as the mean of the percent inhibition of GPa activity ± standard error of the mean (SEM), or the IC_50_ value ± SEM, and represented at least three independent experiments.

### 2.3. The In Vitro Effect of Glucose on Inhibitor Potency

The effects of high levels of glucose on each inhibitor’s potency was evaluated for the most effective compounds. Thus, the effect of increasing glucose concentrations was evaluated for compounds **32**, **33**, **38**, **46**, and the positive control, **CP-91149**. The method was performed as described in Section 2.2, with slight modifications. Briefly, in a 96-well plate, the rabbit mGPa (0.38 U/mL), dissolved in a 50 mM HEPES buffer solution with a pH of 7.2, was exposed to the selected compounds (**32**, **33**, **38**, **46**, and the positive control, **CP-91149**) dissolved in DMSO for 15 min at 37 °C. The enzymatic reaction was then started with the addition of a 50 mM HEPES buffer solution with a pH of 7.2 containing 100 mM KCl, 2.5 mM MgCl_2_, 0.25 mM glucose 1-phosphate, and 0.25 mg/mL glycogen. This buffer was used without glucose, or it was enriched with 5 and 10 mM of glucose and was incubated for 30 min at 37 °C in the reaction mixture. After this incubation time, BIOMOL^®^ Green was added, and the reaction was monitored in a microplate reader (Synergy HT, BioTek Instruments, Winooski, VT, USA) at 620 nm. The results of the in vitro inhibitory activity of GPa corresponded to the absorbance value at 15 min, expressed as the mean of the percent of inhibition of GPa activity ± SEM, or the IC_50_ value ± SEM, and represented at least three independent experiments.

### 2.4. Molecular Docking Calculations and Data Analyses

Initially, human GP Protein Data Bank (PDB) structures were retrieved using uniprot ID P06737. However, there were only three different ligands co-crystallized in the inhibitor binding site (also known as caffeine binding site). Therefore, we decided to extend the query to include additional eukaryotic structures up to 2.5 Å resolution, motivated by the fact that multiple flavonoids are co-crystrallized in non-human structures. We then filtered the retrieved PDBs for the presence of ligands in the inhibitor binding site, using BioPandas, which produced a final list of 27 PDBs. All PDBs refered to either human (*n* = 8) or *Oryctolagus cuniculus* (rabbit) proteins (*n* = 19), and all structures closely aligned with 3DDS (a human structure with the best resolution). Human PDBs showed a maximum root mean squared deviation (RMSD) of 0.46 and 0.48 for human and *O. cuniculus* structures, respectively (PDBIDs listed in Table S1).

These complexes were stripped from all small molecules except for the ligand and five key water molecules. The decision to keep these particular waters came from preliminary docking simulations, where having no waters yielded a very poor reproduction of the experimental ligand poses, which meant a water-free docking protocol could not be properly validated. These five waters were selected by the comparison of the waters within the inhibitor binding site in all human structures of the GPa, from which we determined these five waters to be in a common position in all structures.

All structures then underwent structure preparation using MOE v.2020.0901 [37], where minor issues were found (atoms with fractional occupancies) and fixed. The protein–ligand complexes were then protonated using Protonate3D, the ligands were separated from their corresponding proteins, and both 3D structures were saved in separate files. All ligands were additionally energy minimised in MOE using the EHT:Amber10 forcefield.

First, an initial docking validation step was performed by self- and cross-docking calculations (with all co-crystals for all selected PDBs) using LeDock [38] and AutoDock [39] packages. An exhaustiveness search of 1000 solutions produced in one genetic algorithm was run for LeDock, and 150 poses across 50 genetic algorithm runs (7500 solutions) was run for AutoDock. The optimal docking parameters (the scoring function and the protein 3D structure) were then selected according to the largest amount of experimental ligand poses successfully reproduced (RMSD < 2 Å between experimental and predicted poses).

The optimal docking procedure selected was AutoDock + protein structure 6Y55 [40], which was subsequently used for the docking calculations of the evaluated inhibitors, namely, the styrylpyrazoles (4- and 5-styrylpyrazoles), and the chromone derivatives, namely, the 2-styrylchromones, 2-styrylchromone-related derivatives and flavonoids.

To assess protein–ligand interactions, the top docking poses were submitted to the detection of residue contacts using the docker implementation of PLIP [41]. Images of the compounds and the PDB structures were produced using PyMOL v.1.8.4.0 [42]. Data plots were produced using seaborn, and compound structures were handled and analyzed using BioPandas, fconv, and RDKit. Docking results were handled and trends were analyzed using a Jupyter Notebook workflow.

### 2.5. Statistical Analyses

The in vitro inhibitory effects of the compounds against GPa are expressed as the mean of the percent of inhibition ± SEM, or the IC_50_ value ± SEM. A statistical comparison between the most active compounds was performed using a one-way analysis of variance (ANOVA). Differences were considered to be significant at a *p* value of < 0.05. The IC_50_ value and all the statistical analyses were performed using GraphPad Prism™ (version 6.0, GraphPad Software, San Diego, CA, USA).

## 3. Results

### 3.1. The In Vitro Glycogen Phosphorylase Inhibition

In order to allow for the establishment of a structure–activity relationship study, the inhibitory effects of a panel of different compounds, namely, 4- and 5-styrylpyrazoles, the chromone derivatives (flavonoids, 2-styrylchromones, and 2-styrylchromone-related derivatives) and the positive control, **CP-91149**, were studied against mGPa. The results are shown in Table 1. The selected panel of the compounds was distributed into five groups: 4-styrylpyrazoles (**1**–**9**), 5-styrylpyrazoles (**10**–**15**), 2-styrylchromones (**16**–**29**), 2-styrylchromone-related derivatives (**30**–**35**), and flavonoids (**36**–**52**). In 4-styrylpyrazoles (**1**–**9**), all compounds contained a 2-hydroxyaryl substituent at C-3 of the pyrazole core. In the styryl group, different substitutions were tested, including unsubstituted (**1**), chloro (**2**, **7**), methoxy (**3**), trifluoromethyl (**4**), and nitro (**8**, **9**) substituents. Pyrazoles **5**, **6**, and **7** hold alkyl chains at N-1 of the pyrazole moiety, namely, (CH_2_)_9_CH_3_ at pyrazole **5** and (CH_2_)_11_CH_3_ at pyrazoles **6** and **7**. In 5-styrylpyrazoles (**10**–**15**), four compounds hold a 2-hydroxyphenyl at C-3 of the pyrazole moiety (**10**–**13**) and two compounds hold a phenyl tosylate (phenyl 4-methylbenzenesulfonate) at C-3 of the pyrazole moiety (**14**, **15**). Additionally, two compounds (**10**, **14**) hold an unsubstituted styryl group and the others present chloro (**11**) or methoxy (**12**, **13**, **15**) substituents. Furthermore, different substitutions were assessed at N-1 of the pyrazole moiety, including the phenyl (**12**) and tosyl groups (**13**–**15**). In the third set of compounds, 2-styrylchromones (**16**–**29**), which hold a styryl group at C-2 of the chromone core, nine were hydroxylated (**16**–**24**); one was hydroxylated and methoxylated (**25**), and four were only methoxylated (**26**–**29**). Among the group 2-styrylchromone-related derivatives (**30**–**35**), which display a 4-arylbuta-1,3-dien-1-yl at the C-2 of the chromone core, three compounds were hydroxylated (**30**–**32**), two were hydroxylated and methoxylated (**33**–**34**), and one was only methoxylated (**35**). In the last group of compounds, the flavonoids (**36**–**52**), almost all compounds were hydroxylated (**36**–**50**), one was hydroxylated and methoxylated (**51**) and only one was methoxylated (**52**). The hydroxylated flavonoid **49** also holds an *O*-glucuronide at C-7 of the A ring.

No relevant inhibitory activities were observed, up to the highest tested concentration of 50 µM, for 4-styrylpyrazoles (**1**–**9**) and 5-styrylpyrazoles (**10**–**15**).

Among the 2-styrylchromones (**16**–**29**), compound **23**, bearing hydroxy substituents at C-5 and C-7 of the A ring and a catechol moiety on the B ring, was the most active compound of this group, with an IC_50_ value of 31.7 ± 2.4 μM. Interestingly, comparing 2-styrylchromone **23**, with the compounds **21** and **22**, with the same hydroxylation pattern at the A ring and only one hydroxy substituent at the B ring, or an unsubstituted B ring, respectively, showed a remarkable decrease, to lower than 50%, in the inhibitory activity for both compounds,, at the highest tested concentration of 50 µM. The other hydroxylated 2-styrylchromones, **16**–**20**, and **24**, also exhibited low or no inhibitory activities at the maximum tested concentrations. Within the hydroxylated and methoxylated derivative (**25**) and the methoxylated derivatives only (**26**–**29**), low or no inhibitory activities were observed at the highest tested concentration, such as with 2-styrylchromone **26**, which has methoxy substituents at C-5 and C-7 of the A ring and C-3′ and C-4′ of the B ring, with an inhibitory activity of 42.2 ± 5.6%. Therefore, methoxy substituents seem to be disadvantageous for GPa inhibitory activity in the 2-styrylchromone scaffold.

Within the 2-styrylchromone-related derivatives (**30**–**35**), compound **32**, bearing a hydroxy substituent at C-7 of the A ring and a catechol moiety on the B ring, and compound **33**, with a hydroxy substituent at C-5, a methoxy substituent at C-7 of the A ring, and a catechol moiety on the B ring, were the most active of this group, with an IC_50_ value of 16.7 ± 1.5 μM and 15.9 ± 1.1 μM, respectively. The replacement of methoxy substituents in the 2-styrylchromone-related derivative **32**, led to compounds **34** and **35**, diminishing their inhibitory activities to 51.9 ± 1.8% and 35.8 ± 4.6%, respectively, at the highest tested concentration of 50 µM. The hydroxylated 2-styrylchromone-related derivatives, **30** and **31**, showed inhibitory activities lower than 30% up to the highest tested concentration of 50 µM.

In the flavonoids family (**36**–**52**), five compounds, namely **38**, **46**, **47**, **49**, and **51**, showed remarkable inhibitory activities against GPa, with IC_50_ values between 13.2 ± 1.4 and 23.5 ± 2.9 μM. Hydroxylated flavonoids at the A ring, together with hydroxylations at the C and/or B rings (**37**, **39**–**45**, **48**, and **50**), showed inhibitory activities lower than 30%, with the exception of compound **42**, which displayed an inhibitory activity of 35.7 ± 2.1%, at 50 µM. Flavonoid **36**, with only a hydroxy substituent at C-7 of the A ring, and **52**, methoxylated at C-5, C-6, and C-7 on the A ring, also showed negligible activity (<30%).

### 3.2. In Vitro Effects of Glucose on Inhibitor Potency

As 2-styrylchromone-related derivatives and flavonoids showed remarkable inhibitory activities against GPa, two effective compounds of each group were selected (**32**, **33**, **38**, **46**) in order to evaluate the influence of the glucose levels of the inhibitory activities. The positive control, **CP-91149**, was also evaluated. The results are represented in Table 2, showing the obtained IC_50_ in the absence of glucose, and at 5 mM, and 10 mM glucose concentrations.

Interestingly, the inhibitory potencies of compounds **32**, **38**, **46**, and the positive control, **CP-91149**, increased in the presence of glucose levels at 5 mM and 10 mM. Particularly, the flavonoid **46**, norwogonin, showed an IC_50_ value of 13.2 ± 1.4 µM in the absence of glucose, which decreased to an IC_50_ of 3.7 ± 0.5 µM in the presence of 10 mM of glucose. Moreover, at 10 mM of glucose, the flavonoid **46** and the positive control, **CP-91149**, presented no statistical differences between them, as shown by the ANOVA analysis. The 2-styrylchromone-related derivative **33** was the only compound that displayed a different behavior, decreasing the inhibitory activity with an increase in the glucose concentration.

### 3.3. Molecular Docking Studies

**Figure 3:**
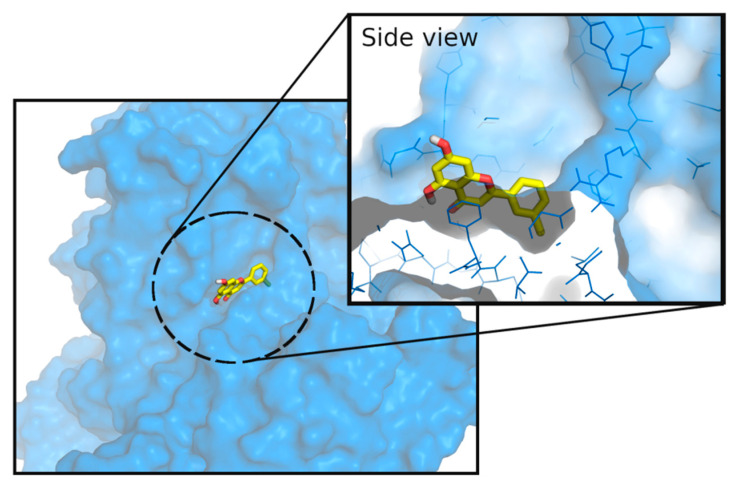
X-ray structure of GPa protein (from 6Y55) in blue, and its co-crystallised ligand in yellow. The ligand is bound at the inhibitor binding site (or caffeine binding site).

GP displays several different binding sites, including the catalytic site, the allosteric or adenosine monophosphate (AMP) binding site, the new allosteric binding site, the inhibitor site, the purine or caffeine site, the glycogen storage site, and the quercetin binding site [36]. Molecular docking studies were performed in the inhibitor binding site (Figure 3).

The molecular docking protocol for GPa docking calculations was validated through self- and cross-docking experiments. We observed that nine ligands with AutoDock and twelve ligands with LeDock were well reproduced (RMSD < 2.5 Å) in their original PDB structures (Figure S1). The PDBs that showed a good self-docking performance was submitted to cross-docking, where we considered only the ligands with structures that were more representative of the compounds in this work (1L5R, 3EBO, 1C8K, 1Z62, 3EBP, 6Y55, 6Y5C, 6Y5O). We noticed that AutoDock performed significantly better than LeDock and, overall, 6Y55 paired with AutoDock reached the best overall performing conditions (Figure S1), which was evident by the largest number of X-ray ligands reproduced within 2 Å of their experimental poses (*n* = 8).

After this preliminary validation, docking calculations were performed for all the families of potential GPa inhibitors studied in this work; namely, the styrylpyrazoles (4- and 5-styrylpyrazoles), and the chromone derivatives; namely, the 2-styrylchromones, 2-styrylchromone-related derivatives, and flavonoids. Molecular docking studies were also performed for the positive control, **CP-91149**, to enable comparisons with the studied compounds.

**Figure 4:**
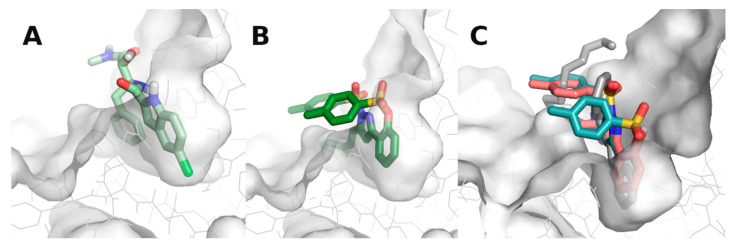
Styrylpyrazoles in GPa’s inhibitor binding site. (**A**) **CP-91149** (pale green) in the inhibitor binding site. (**B**) Compound **14** (dark green) in the inhibitor binding site. (**C**) Compounds **6** (grey), **13** (salmon), and **15** (light teal).

Docking results for the styrylpyrazoles showed that some of the compounds had a very similar interaction profile compared to **CP-91149** (Figure S2), e.g., compound **6**, **13**, and **14** (Figure 4). However, these compounds also showed non-polar groups facing away from the binding pocket, occupying what is likely to be a region populated with water molecules. All other styrylpyrazoles failed to establish two of the three key interactions (an H-bond with HIS614 and a water bridge with MET615) seen with **CP-91149**. It is worth noting that many of the styrylpyrazoles were unable to properly occupy the most buried portion of the pocket (the bottom of the “V”).

**Figure 5:**
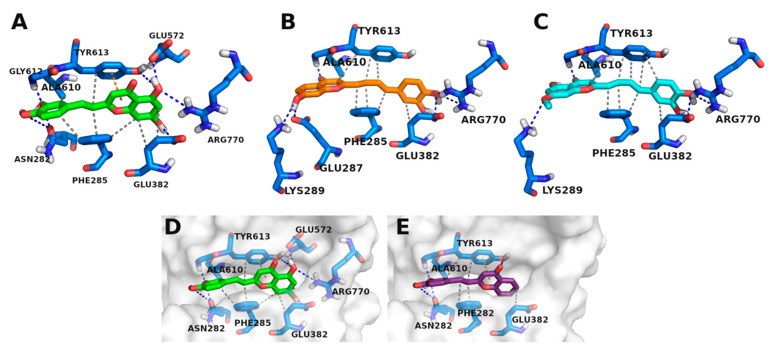
Placement of 2-styrylchromones and 2-styrylchromone-related derivatives in the inhibitor binding site of GPa. (**A**) Compound **23** (green) and the GPa residues it interacts with. (**B**) Compound **32** (orange) and the GPa residues it interacts with. (**C**) Compound **33** (cyan) and the GPa residues it interacts with. (**D**) Compound **23** (green) versus (**E**) compound **18** (purple) in the inhibitor binding site. Bond types are encoded as follows: hydrophobic interactions (grey), hydrogen bonds (blue), pi-stacking (orange).

The docking results for 2-styrylchromones and 2-styrylchromone-related derivatives showed that the three active compounds, **23**, **32**, and **33**, interacted with the two extreme ends of the pocket; namely, ASN282 or LYS289, which were paired with a simultaneous contact with GLU382 (Figure 5), in contrast to other compounds, e.g., **21** and **22**, which had just one or no H-bonding groups in the B ring (Figure S3). 2-Styrylchromone **25** revealed that an H-bonding donor, specifically, was required in the A ring, as this compound had an H-bond acceptor at C-7 of the A ring. This was ideal for reaching GLU382, but it was not able to establish an H-bond with the H-acceptor carboxylate in the GLU382 side chain. A similar situation was observed with the 2-styrylchromones **26**, **27**, **28**, and **29**, in which the compounds rotated, positioning the B ring near GLU382 (possibly, the A ring became too bulky to fit in this narrower end of the pocket). As seen with 2-styrylchromone **25**, these four compounds also had a H-bonding group near GLU382 (C-3′ of the B ring). However, the H-bonding with this residue was not possible, as these were all H-acceptors. The requirement for these two simultaneous points of contact was demonstrated by cases such as that of 2-styrylchromone **18**, which perfectly aligned with **23**. However, the former had no H-bonding groups at the end of the two fused rings (A and C), and was, therefore, not able to establish any H-bonding near LYS289.

2-Styrylchromone-related derivatives **30** and **31** aligned perfectly with the active compound **32**. However, they were unable to establish an H-bond with LYS289, due to the lack of the H-bonding group that **32** had at C-7 of the A ring. The only exception to this ASN282/LYS289 + GLU382 pattern was compound **34**, which established this set of interactions, despite being less active (51.9 ± 1.8% 50 μM). Compound **34** was perfectly aligned with the active compound **33**, differing only by a single hydroxy substituent in C-5 of the A ring, which was present in the 2-styrylchromone-related derivative **33**.

**Figure 6:**
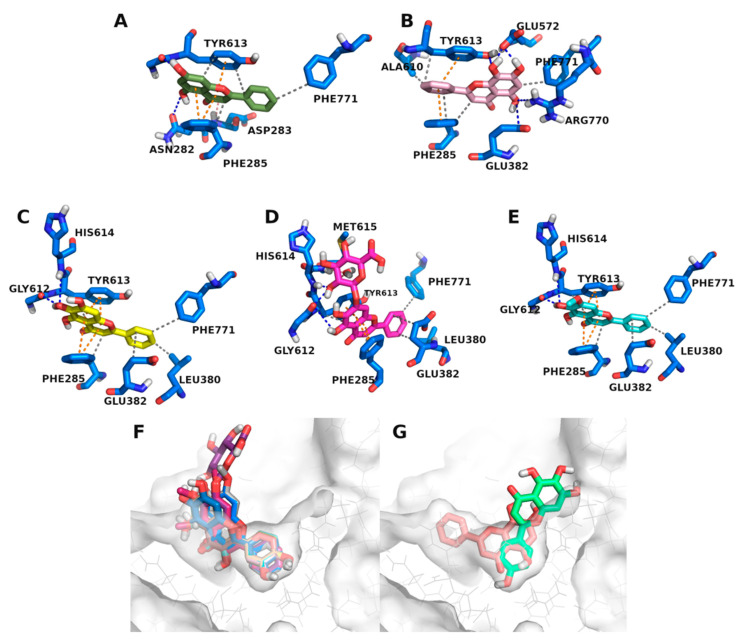
Placement of flavonoids in the GPa inhibitor binding site. (**A**) Contacts established by compound **38** (dark green). (**B**) Contacts established by compound **46** (light pink). (**C**) Contacts established by compound **47** (yellow). (**D**) Contacts established by compound **49** (magenta). (**E**) Contacts established by compound **51** (pale blue). (**F**) All flavonoids, except **46** and **48**, inside the GPa inhibitor binding site, versus (**G**) **46** (light pink) and **48** (turquoise). Bond types are encoded as follows: hydrophobic interactions (grey), hydrogen bonds (blue), pi-stacking (orange).

Finally, the flavonoids family was also docked into the GPa inhibitor binding site. We found that compound **46** (Figure 6), which was one of the most effective in the flavonoid family, was the only compound in this series with the simultaneous H-bonding with two residues on either side of the longitudinal plane of the pocket, in contrast to the remaining compounds (Figure S4). All other flavonoids, except for compound **48**, were positioned in an inverted placement compared to that of compound **46**, in a close alignment, with the B ring occupying the vertex of this V-shaped pocket. This indicated a second binding mode that was possible for the flavonoid scaffold. Overall, the binding profile for the active compounds in this series indicated that they possibly bound to the inhibitor binding site, given their close resemblance (in terms of their placement in the pocket) to different flavonoids co-crystallized with GPa.

We did not find a perfect correlation between docking scores (produced by docking calculations) and GPa inhibitory activities (Figure S5 and Table S2). The same behavior had also been observed for ChEMBL compounds (Figure S6). However, the analyses on the docked potential of the GPa–inhibitor interaction patterns and poses, together with the comparison with the interactions made between the protein and its X-ray ligands, always enabled the identification of groups and their interactions, that could account for activities, or a lack thereof.

## 4. Discussion

Glycogenolysis is an important pathway in hepatic glucose production, frequently exacerbated in type 2 DM patients [16]. Diverse compounds have been studied as GP inhibitors [10,13,16] to target and control glycemia. However, despite multiple studies, the results in the literature are not always comparable due to the existence of several differences among the experimental conditions. Particularly, several sources of the enzyme (human, pig, rabbit, or rodent), with different isoforms in both the phosphorylated and dephosphorylated states have been used. Moreover, due to the fact that this enzyme catalyzes a reversible reaction, different activity measurement methods have been employed [43,44]. The present study overcomes these discrepancies by assessing a panel of 52 structurally related compounds against GPa activity, using an optimized and validated method [36]. The commercial rabbit muscle isoform of GP was used in this study. As already mentioned, the brain, liver, and muscle isoforms of GP share an amino acid sequence homology of nearly 80%. Furthermore, the catalytic site of GP in mammalian species is highly conserved, sharing a homology of nearly 100% at this site. Therefore, the compounds inhibiting mammalian forms at this site, in principle, are also able to inhibit human lGP [16]. The phosphorylated form of this enzyme, GPa, was chosen for the present study, since this form represents the catalytically active one.

Different families of compounds were evaluated against GPa, including styrylpyrazoles, namely, 4- (**1**–**9**) and 5-styrylpyrazoles (**10**–**15**), and different chromone derivatives, namely, 2-styrylchromones (**16**–**29**), 2-styrylchromone-related derivatives (**30**–**35**), and flavonoids (**36**–**52**). To support the in vitro experimental findings and enable the rationalization of the obtained structure–activity relationships, docking studies for all the 52 compounds were also performed. The docking protocol (using 6Y55′s X-ray structure and the open-access software AutoDock) showed an excellent ability to predict the X-ray poses of all X-ray ligands available in the inhibitor binding site of GPa (RMSD < 2.5 Å).

The first family of compounds, the 4- (**1**–**9**) and 5-styrylpyrazoles (**10**–**15**), were studied here for the first time. The styrylpyrazoles (**1**–**15**) showed no inhibitory activities against GPa, up to the maximum tested concentration of 50 μM. As far as we know, only one study has been conducted using 5-(1-aryl-1*H*-pyrazol-3-yl)-1*H*-tetrazoles against human lGP inhibitors in an in silico study. The authors reported that the most potent pyrazoles exhibited good docking scores against GP. However, in vitro studies with the isolated enzyme were not conducted [45]. Our docking studies showed that this series of compounds shared some resemblance to the positive control, **CP-91149**. Some of the styrylpyrazoles established a very similar interaction profile to **CP-91149**, such as **14**. Despite having a π-stack with PHE285, an H-bond with HIS614, and a water bridge with MET615, the styrylpyrazole **14** had two *p*-methylphenyl groups that were highly exposed to the solvent. It is possible that these non-polar groups were able to be positioned in a docking calculation in which most waters were removed. However, considering that this was a highly solvent-exposed pocket, we hypothesise that the presence of these large non-polar groups might make the binding energetically non-viable. The styrylpyrazoles **6**, **13**, and **14** also showed a very similar binding profile to **CP-91149**; however, similar to **14**, these compounds displayed non-polar groups facing away from the binding pocket and occupying what was likely to be a region populated with water molecules. All other styrylpyrazoles were likely inactive because they failed to establish two of the three key interactions (an H-bond with HIS614 and a water bridge with MET615) seen with **CP-91149**, which might explain a low level of activity owed to weak binding. It is worth noting that many of the styrylpyrazoles were unable to properly occupy the most buried portion of the pocket (the bottom of the “V”).

In the case of the 2-styrylchromones (**16**–**29**), the hydroxy and methoxy substituents were evaluated. 2-Styrylchromone **23** was the most active of the group, where the presence of the hydroxy substituents at C-5 and C-7 of the A ring, and a catechol moiety on the B ring, contributed to the inhibitory activities against GPa. When comparing 2-styrylchromone **23** with the compounds **16**–**22**, it is possible to conclude that the addition of the hydroxy substituents increased the inhibitory activity of the 2-styrylchromones. The comparison between 2-styrylchromones **23** and **21**, holding hydroxy substituents at C-5 and C-7 of the A ring, provided evidence for the positive effect of the catechol moiety on the B ring (**23**), which favored the inhibitory effect against GPa. However, the presence of the catechol moiety was only effective when the hydroxy substituents were present at C-5 and C-7 of the A ring, as observed by the comparison of 2-styrylchromone 23 with 19 and 20. The comparison of 2-styrylchromones **23** and **26**, bearing methoxy substituents at the same positions as the hydroxy substituents in **23**, allowed the conclusion that the presence of methoxy substituents in the 2-styrylchromone scaffold did not provide any advantage for the intended effect. When comparing 2-styrylchromone **23** with **25**, holding methoxy substituents at C-5 and C-7 of the A ring and a catechol moiety on the B ring, also enabled the verification of the importance of the hydroxy substituents at C-5 and C-7 of the A ring, together with the catechol moiety, and the disadvantageous effects of methoxy substituents for the inhibitory activity. As far as we know, 2-styrylchromones were studied here for the first time.

Among the 2-styrylchromone-related derivatives (**30**–**35**), good inhibitory activities were observed, showing IC_50_ values lower than 20 µM. The 2-styrylchromone-related derivative **32**, which holds a hydroxy substituent at C-7 of the A ring and a catechol moiety on the B ring, and the 2-styrylchromone-related derivative **33,** with the same substitutions at the B ring, a hydroxy substituent at C-5, and a methoxy substituent at C-7 of the A ring, were the most active compounds of the group. 2-Styrylchromone **19**, which is structurally related to the 2-styrylchromone-related derivative **32**, was not able to inhibit GPa at the maximum tested concentration. Interestingly, the 2-styrylchromone **19**, with one hydroxy group at the A ring and the catechol at the B ring, did not show any effects in contrast with the structurally related 2-styrylchromone-related derivative **32**. The change of the hydroxy substituent at the A ring from C-7 (**32**) to C-5 (**31**) led to a complete loss of activity at the highest tested concentration of 50 µM. However, the addition of a methoxy substituent to C-7 in the 2-styrylchromone-related derivative **31,** resulted in a huge improvement in the inhibitory activity in the 2-styrylchromone-related derivative **33**. Still, when comparing the 2-styrylchromone-related derivative **32** with **34** and **35**, the presence of methoxy substituents disfavored the inhibitory activity. To the best of our knowledge, the inhibitory activity of these 2-styrylchromone-related derivatives were not tested previously for GP inhibitory activities.

Docking studies revealed that among the 2-styrylchromones and 2-styrylchromone-related derivatives, three active compounds (**23**, **32**, and **33**) were found and showed a characteristic binding pattern that separated them from the remaining compounds. These three compounds appeared to require H-bonding with two extreme ends of the pocket, ASN282 or LYS289, paired with a simultaneous contact with GLU382. This was partly in line with observations from X-ray ligands which showed frequent contact with ASN282. This was shown by comparing different sets of active and inactive compounds which were closely aligned. For instance, the inactive 2-styrylchromone **18** lacked H-bonding groups at the extremities of the A and C rings, making it unable to interact with LYS289, compared to 2-styrylchromone **23**. In the same way, despite having one H-donor at the C-7 position of the A ring, the 2-styrylchromone **19** deviated towards ASN284. This is likely due to a lack of additional H-bonding groups closer to the carbonyl at the A ring, which were able to anchor the compound closer to the bottom of the V-shaped pocket. 2-Styrylchromone **20** had the opposite problem, having a hydroxy substituent at C-5 of the A ring but no additional H-bonding groups that could bind to GLU382. Other compounds such as the 2-styrylchromones **21** and **22** had just one or no H-bonding groups in the B ring. Therefore, different sets of compounds revealed that an H-bond donor in the A ring was needed to mediate bonding with GLU382 and H-bonding between the C-7 position of the A ring and LYS289, which was determinant for the inhibitory activity of the compounds. It should be noted that one compound showed this dual-point H-bonding pattern, while still showing reduced inhibitory activity (**34**, with 51.9 ± 1.8% at 50 μM). This indicates that this rule appears to be required, but not sufficient. Perhaps additional derivatives in this series would uncover a second pattern of binding, or a more complex pattern, than the indicated in this study. Nevertheless, the 2-styrylchromone-related derivative **34** perfectly aligned with the 2-styrylchromone-related derivative **33**, differing only by an additional hydroxy substituent at C-5 of the A ring in the 2-styrylchromone-related derivative **33**, which did not establish any bonds with the binding site. This indicates that this group appears to play no role in the binding of the 2-styrylchromone-related derivative **33**. However, it is highly likely that in biological conditions, it helps to stabilize the compound in the binding site by establishing water bridges. This hypothesis stems from the fact that this pocket was densely surrounded by water molecules, most of which were excluded from the calculation to avoid restricting the poses of compounds that were structurally different from the native X-ray ligand.

2-Styrylchromones (**16**–**29**) share the styryl group backbone as displayed in the 4-(1–9) and 5-styrylpyrazoles (**10**–**15**). However, no relevant inhibitory activities were found in pyrazole groups. Comparing these families of compounds, possibly, the existence of a catechol in the styryl moiety, together with hydroxy substituents in the 2-hydroxyaryl substituent at the C-3 of the pyrazole moiety, could benefit the inhibitory activity of pyrazoles.

Regarding the flavonoid (**36**–**52**) group, five compounds, namely **38**, **46**, **47**, **49**, and **51**, showed remarkable inhibitory activities against GPa, with IC_50_ values lower than 24 µM. Flavonoids are well-known inhibitors of GP [40,46,47,48,49]. However, their use as GP inhibitors and their binding sites have not yet been fully explored. In this study, a series of hydroxylated flavonoids (**36**–**50**) were evaluated, together with one hydroxylated and methoxylated flavonoid (**51**) and one methoxylated flavonoid (**52**). Among the most active compounds **38**, **46**, **47**, **49**, and **51**, flavonoids **38**, **46**, and **47** were only hydroxylated at the A ring, while flavonoid **49** was hydroxylated and also held an *O*-glucuronide at C-7 of the A ring. Flavonoid **51** was hydroxylated and methoxylated at the A ring.

Flavonoid **38**, chrysin, was one of the most active compounds in this study and it is recognized as a potent GP inhibitor by several authors [10,46,47,50], corroborating our results. Chetter et al. [40] studied in silico and in vitro, a panel of flavonoids using chrysin as the core scaffold against rabbit mGPa and mGPb, together with human lGPa. In the in vitro studies, performed in the direction of glycogen synthesis, the flavonoid **38**, chrysin, was more potent than the flavonoid **45**, quercetin, by almost 10-fold [40], which is in agreement with our results. Nonetheless, Kato et al. [48] reported an IC_50_ value higher than 200 µM for the flavonoid **38**, chrysin [48]. These dissimilar results could be explained by the differences found in the experimental conditions.

When comparing flavonoid **36**, which has a hydroxy substituent at C-7 of the A ring, with flavonoid **38**, chrysin, which holds hydroxy substituents at C-5 and C-7, it is possible to verify that the addition of the hydroxy substituent at C-5, increased GPa inhibitory activity. Additionally, the comparison of flavonoid **38** with flavonoids **46** and **47,** with the addition of hydroxy groups at the A ring at C-8 and C-6, respectively, maintained the inhibitory activity. Jakobs et al. [47] studied some naturally occurring flavonoids in the activity of isolated rabbit mGPa and mGPb, in the direction of the glycogen breakdown. The authors reported that the flavonoids **38**, chrysin, and **47**, baicalein, inhibited rabbit mGPa (IC_50_ values of 27.5 µM and 11.2 ± 1.5 µM, respectively) and GPb (IC_50_ values of 15.3 ± 1.0 µM and 10.2 ± 1.2 µM, respectively), similar to our results. The authors established some structural requirements for GP inhibition, reporting the satisfactory inhibitory effects with flavonoids **38**, chrysin, and **47**, baicalein, which hold hydroxy substituents at C-5 and C-7 of the A ring and three adjacent hydroxy groups at C-5, C-6, and C-7 of the A ring, respectively [47]. The same effects were observed in our results. Recently, Brás et al. [46] assessed the inhibitory activity of some flavonoid derivatives against the inhibitor site of rabbit mGP, using an in silico approach. Corroborating our results, the authors suggested that three adjacent hydroxy groups at the A ring, mainly the hydroxy groups at C-5 and C-7 of the A ring, with an unsubstituted B ring, seemed sufficient for an effective inhibitory potency [46].

In flavonoids with additional hydroxy substituents at the B and C rings, such as flavonoid **42**, apigenin, which has hydroxy substituents at C-5 and C-7 of the A ring and at C-4′ of the B ring, and flavonoid **44**, galangin, which holds hydroxy substituents at the same positions of the A ring as **42**, but with an additional one at C-3 of the C ring, a loss of the inhibitory activity at the highest tested concentration of 50 µM is observed. The same effect is observed when flavonoid **47**, baicalein, is compared with flavonoids **48**, scutellarein, and **50**, quercetagetin. Therefore, the hydroxylation of the A ring and absence of substituents at the B and C rings is crucial for the inhibitory activity of flavonoids. Jakobs et al. [47] described the importance of a hydroxy substituent at C-3 of the C ring and the vicinal hydroxy substituents at the B ring [47]. These conclusions were not in accordance with our results. Brás et al. [46] reported that the presence of a hydroxy substituent at C-3 of the C ring was not essential for the inhibitory activity. However, the hydroxylation at C-4′ of the B ring could be important [46], which was not observed in our results. In our study, the hydroxylation at the C-4′ position of the B ring led to a decrease in inhibitory activity, as observed by the comparison of the flavonoids **38** and **42**.

The docking results for the flavonoid family showed that establishing four simultaneous H-bonds, two in either “wall” of the pocket, drove activity. This was evident in flavonoid **46**, norwogonin, which was one of the most active in this family, and the only one where this effect was observed. GLU572 was the additional residue with which only flavonoid **46** interacted, and, notably, two H-bonds were established between this residue and the compound. The inspection of the pose showed that the presence of two hydroxy substituents at C-7 and C-8 of the A ring was a key determinant of the binding to GPa’s inhibitor binding site. The two consecutives hydroxy substituents aligned the compound with GLU572′s carboxyl, which, in turn, placed the hydroxy substituent at C-5 of the A ring, in an ideal H-bond distance to GLU372 and ARG770. The latter was also found in one of the X-ray ligands (1L5R, Figure S7).

An inspection of the poses of active and inactive compounds revealed that an H-bond donor that was simultaneously in C-5 and C-7 of the A ring, paired with a no H-bonding group in the B ring, was likely a strong determinant of activity. This was evident by the fact that multiple inactive flavonoids were perfectly overlaid with derivative **38**, chrysin (i.e., **36**, **37**, **39**, **40**, **41**, **42**, **43**, **45**, **50**), but either lacked a hydroxy substituent at C-5 of the A ring or in both C-5 and C-7 of the A ring. This specific placement of hydroxy substituents allowed the dual H-bonding with GLY612 and ASN282 (these two simultaneous interactions occurred in several X-ray ligands). One exception was flavonoid **44**, galangin, which, despite having a very similar pose to flavonoid **38**, chrysin, as well as fulfilling this binding pattern, was inactive. Inspecting the pose of the two compounds showed that the additional hydroxy substituent at C-3 on the C ring in compound **44**, galangin, might be anchoring to ASP283′s backbone carbonyl (via an H-bond), restricting the compound to correctly align the A and C rings between PHE285 and TYR613 to establish multiple π-stacking interactions. ASP283 already established a water bridge with both compounds, and the additional H-bond might dramatically reduce the mobility of the compound inside the GPa inhibitor binding site.

Alternatively, it is possible for a compound to remain active (albeit typically lower) without an H-bond donor at C-7 of the A ring, as long as it has an H-donor group close by, for example, at C-6 of the A ring, and complies with the rest of the binding pattern described above. This was the case of the flavonoids **47**, **49**, and **51**. The H-donor in the C-6 of the A ring bound to GLY612, replacing the role of an H-donor in the C-7 position of the A ring (which bound to this residue as well). Interestingly, this replacement of C-7 to C-6 rotated the compound slightly upwards (as C-6 took the space C-7 would occupy), which created a possibility for an additional bridge with a water molecule. It should be noted that among all compounds in this study, flavonoid **46**, norwogonin, was the only compound that was able to establish a double H-bond with GLU572.

Interestingly, opposite effects were observed in the structurally related 2-styrylchromone (**16**–**29**) and 2-styrylchromone-related derivative (**30**–**35**) groups, for which the presence of additional hydroxy groups at the B ring was crucial for the inhibitory activity of the compounds.

The methoxylation of flavonoid **51**, and the glycosylation of flavonoid **49**, were not disadvantages for the effect under study. However, the complete methoxylation of flavonoid **48**, observed in compound **52**, led to a complete loss of activity at the highest tested concentration of 50 µM. Similarly, this effect was also correlated with 2-styrylchromones (**16**–**29**) and 2-styrylchromone-related derivatives (**30**–**35**). Therefore, in general, methoxylations are not favorable for the intended effect. This conclusion is corroborated by other authors [47,48].

Hypoglycemia is a common adverse effect in patients with DM who are mainly treated with insulin and insulin secretagogues. Patients with hypoglycemia are not able to achieve normal plasma glucose concentrations. Therefore, hypoglycemia is one of the most limiting factors of DM treatments and a major limiting factor on the quality of life of DM patients. This condition can be fatal and seems to be correlated with an increased risk of developing cardiovascular disease and subsequent mortality [51]. Therefore, developing novel approaches in DM treatment is crucial. One of the major advantages of GP inhibitors is their ability to inhibit the enzyme at elevated glucose levels, decreasing the inhibitory potential when blood glucose is lowered. Therefore, the risk of developing hypoglycemia is reduced [7]. The present work assessed the effect of higher levels of glucose, mimicking the hyperglycemia condition, on the inhibitory activity of the selected compounds. Two effective compounds of the 2-styrylchromone-related derivative and flavonoid groups were selected, namely, **32**, **33**, **38**, and **46**, together with the positive control, **CP-91149**. Interestingly, the IC_50_ value of these compounds, except the 2-styrylchromone-related derivative **33**, decreased when the concentration of glucose increased. The 2-styrylchromone-related derivative **33** exhibited an opposite behavior, not acting synergically with glucose. The results for the positive control, **CP-91149**, are in agreement with the results previously reported by Martin et al. [52]. Notably, the IC_50_ value of the flavonoid **46**, norwogonin, decreased almost four-fold when the concentration of glucose increased to 10 mM, showing no statistical differences when compared with the positive control, **CP-91149**. Therefore, flavonoid **46**, norwogonin, was considered the most active compound of this study, due to a similar effect with the positive control, **CP-91149**.

Flavonoids, the most common type of polyphenols in the human diet and the most widely distributed in nature, demonstrated promising inhibitory effects against GP, as demonstrated here for the flavonoid **46**, norwogonin. The bioavailability of flavonoids and their metabolites have been an important topic of research. Their potential biological activities in vivo depend on their absorption, distribution, metabolism, and excretion (ADME). Despite the poor bioavailability and high metabolization of some flavonoids in vivo, several options need to be implemented to increase the beneficial therapeutic effects of these agents, including the use of nanotechnology [25].

## 5. Conclusions

**Figure 7:**
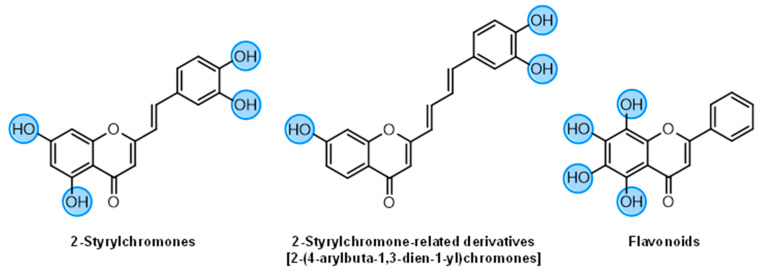
Structure–activity relationship of chromone derivatives: 2-styrylchromones, 2-styrylchromone-related derivatives [2-(4-arylbuta-1,3-dien-1-yl)chromones], and flavonoids, which contributed to increased GP inhibitory activity.

In the present study, a panel of 52 compounds, including chromone derivatives (flavonoids, 2-styrylchromones, and 2-styrylchromone-related derivatives) and styrylpyrazoles (4- and 5-styrylpyrazoles), were evaluated for their inhibitory activities against GPa, the key enzyme in the glycogenolysis pathway. While the tested styrylpyrazoles were not able to inhibit GPa, at the maximum tested concentrations, several of the tested compounds from the other families showed an effective inhibitory effect. It was observed that GPa inhibition depends on the substitution pattern of the compounds. In 2-styrylchromone and 2-styrylchromone-related derivative groups, hydroxylations at the A and B rings were crucial for the inhibitory activity; in the flavonoid group, the hydroxylation of the A ring was determinant for the inhibitory activity (Figure 7). A molecular docking analysis showed that some styrylpyrazoles share some similarity in poses and interactions with residues at the GPa inhibitor binding site with the positive control, **CP-91149**. When interacting with PHE285, HIS614, and MET615, these compounds exhibited non-polar groups facing away from the inhibitor site to a region occupied by the solvent. The remaining styrylpyrazoles failed to establish at least two of the three key interactions (PHE285, HIS614, and MET615). 2-Styrylchromone and 2-styrylchromone-related derivative docking poses in the GPa inhibitor binding site showed that the active compounds of this family should have a characteristic binding pattern, requiring H-bond interactions with the two extreme ends of the pocket (ASN282/LYS289 and GLU382) to display activity. The flavonoid family showed two possible binding modes. Flavonoid **46**, norwogonin, showed a unique binding mode in this series, interacting with both sides of the longitudinal plane of the pocket (H-bonds with two residues in each side). This is the only tested compound that interacts with GLU572, suggesting that the presence of two hydroxy substituents at C-7 and C-8 may be critical for the activity. The inactive compounds in this series never established H-bonds with ALA610, HIS614, and MET615, but interacted with HIS571 and TYR573.

The inhibitory activity of the flavonoid **46**, norwogonin, increased in the presence of high glucose concentrations, with a similar effect to the positive control, **CP-91149**. This outcome could reduce the risk of developing hypoglycemia, a common reported adverse effect associated with the use of insulin and insulin secretagogues in DM treatment.

This work provides important considerations and a better understanding of novel potential scaffolds for the study of novel GP inhibitors.

## Figures and Tables

**Table 1:** Chemical structures and in vitro inhibitory activities of styrylpyrazoles, namely, 4- and 5-styrylpyrazoles, as well as the chromone derivatives 2-styrylchromones, the 2-styrylchromone-related derivatives [2-(4-arylbuta-1,3-dien-1-yl)chromones], and flavonoids against GPa.

**4-Styrylpyrazoles** 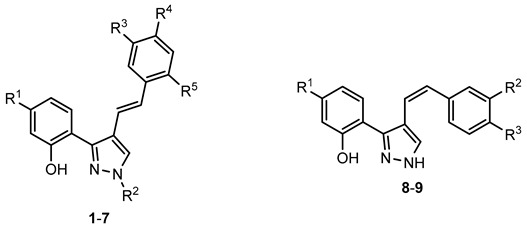 **(1–9)**								
**Compounds**	**R^1^**		**R^2^**		**R^3^**	**R^4^**	**R^5^**	**GPa inhibitory activity (%) or IC_50_ value**
**1**	-		-		-	-	-	<30% ^50 μM^
**2**	-		-		-	Cl	-	<30% ^50 μM^
**3**	-		-		-	OCH_3_	-	<30% ^50 μM^
**4**	-		-		-	-	CF_3_	<30% ^50 μM^
**5**	-		(CH_2_)_9_CH_3_		-	-	-	<30% ^50 μM^
**6**	-		(CH_2_)_11_CH_3_		-	-	-	<30% ^50 μM^
**7**	-		(CH_2_)_11_CH_3_		-	Cl	-	<30% ^50 μM^
**8**	-		NO_2_		-	-	-	<30% ^50 μM^
**9**	OCH_3_		-		NO_2_	-	-	<30% ^50 μM^
**5-Styrylpyrazoles** 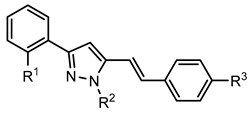 **(10–15)**								
**Compounds**	**R^1^**		**R^2^**			**R^3^**		**GPa inhibitory activity (%) or IC_50_ value**
**10**	OH		-			-		<30% ^50 μM^
**11**	OH		-			Cl		<30% ^50 μM^
**12**	OH		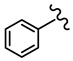			OCH_3_		<30% ^50 μM^
**13**	OH		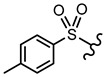			OCH_3_		<30% ^50 μM^
**14**	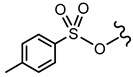		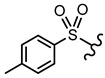			-		<30% ^50 μM^
**15**	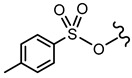		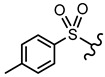			OCH_3_		<30% ^50 μM^
**2-Styrylchromones** 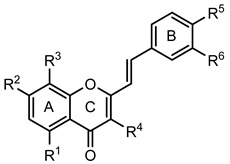 **(16–29)**								
**Compounds**	**R^1^**		**R^2^**	**R^3^**	**R^4^**	**R^5^**	**R^6^**	**GPa inhibitory activity (%) or IC_50_ value**
**16**	-		OH	-	-	-	-	<30% ^50 μM^
**17**	-		OH	-	-	OH	-	<30% ^50 μM^
**18**	-		-	-	-	OH	OH	<30% ^50 μM^
**19**	-		OH	-	-	OH	OH	<30% ^50 μM^
**20**	OH		-	-	-	OH	OH	<30% ^50 μM^
**21**	OH		OH	-	-	-	-	30.1 ± 6.1 % ^50 μM^
**22**	OH		OH	-	-	OH	-	43.2 ± 5.1 % ^50 μM^
**23**	OH		OH	-	-	OH	OH	**31.7 ± 2.4 µM** ^(b)^
**24**	-		OH	OH	OH	OH	OH	33.7 ± 6.5% ^25 μM^
**25**	OCH_3_		OCH_3_	-	-	OH	OH	<30% ^50 μM^
**26**	OCH_3_		OCH_3_	-	-	OCH_3_	OCH_3_	42.2 ± 5.6% ^50 μM^
**27**	-		OCH_3_	OCH_3_	-	OCH_3_	OCH_3_	<30% ^50 μM^
**28**	-		OCH_3_	-	-	OCH_3_	OCH_3_	<30% ^50 μM^
**29**	OCH_3_		-	-	-	OCH_3_	OCH_3_	<30% ^50 μM^
**2-Styrylchromone-related derivatives** **[2-(4-arylbuta-1,3-dien-1-yl)chromones]** 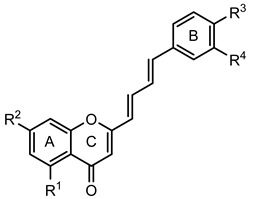 **(30–35)**								
**Compounds**	**R^1^**		**R^2^**		**R^3^**		**R^4^**	**GPa inhibitory activity (%) or IC_50_ value**
**30**	-		-		OH		OH	<30% ^50 μM^
**31**	OH		-		OH		OH	<30% ^50 μM^
**32**	-		OH		OH		OH	**16.7 ± 1.5 µM** ^(c)^
**33**	OH		OCH_3_		OH		OH	**15.9 ± 1.1 µM** ^(c)^
**34**	-		OCH_3_		OH		OH	51.9 ± 1.8% ^50 μM^
**35**	-		OCH_3_		OCH_3_		OCH_3_	35.8 ± 4.6% ^50 μM^
**Flavonoids** 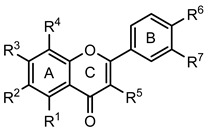 **(36–52)**								
**Compounds**	**R^1^**	**R^2^**	**R^3^**	**R^4^**	**R^5^**	**R^6^**	**R^7^**	**GPa inhibitory activity (%) or IC_50_ value**
**36**	-	-	OH	-	-	-	-	<30% ^50 μM^
**37**	-	-	OH	-	-	OH	-	<30% ^50 μM^
**38** Chrysin	OH	-	OH	-	-	-	-	**19.3 ± 1.5 µM** ^(c)^
**39**	-	-		-	-	OH	OH	<30% ^50 μM^
**40**	-	-	OH	-	-	OH	OH	<30% ^50 μM^
**41**	OH	-		-	-	OH	OH	<30% ^50 μM^
**42** Apigenin	OH	-	OH	-	-	OH	-	35.7 ± 2.1% ^50 μM^
**43** Luteolin	OH	-	OH	-	-	OH	OH	<30% ^50 μM^
**44** Galangin	OH	-	OH	-	OH	-	-	<30% ^50 μM^
**45** Quercetin	OH	-	OH	-	OH	OH	OH	<30% ^50 μM^
**46** Norwogonin	OH	-	OH	OH	-	-	-	**13.2 ± 1.4 µM** ^(c)^
**47** Baicalein	OH	OH	OH	-	-	-	-	**23.5 ± 2.9 µM** ^(b, c)^
**48** Scutellarein	OH	OH	OH	-	-	OH	-	<30% ^50 μM^
**49** Baicalin	OH	OH	O-Glu curoni de	-	-	-	-	**20.5 ± 2.5 µM** ^(c)^
**50** Quercetagetin	OH	OH	OH	-	OH	OH	OH	<30% ^50 μM^
**51** Baicalein-7-methylether	OH	OH	OCH_3_	-	-	-	-	**22.6 ± 0.4 µM** ^(b, c)^
**52** Baicalein-5,6,7-trimethylether	OCH_3_	OCH_3_	OCH_3_	-	-	-	-	<30% ^50 μM^
**Positive** **control** **CP-91149**	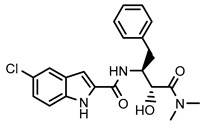							**0.58 ± 0.09 μM** ^(a)^

Mean of the percent inhibition ± SEM represents at least three independent experiments. Concentrations shown in superscript indicate the highest concentrations that could be tested without interferences with the methodology. The IC_50_ values (represented in bold) with different lowercase superscript letters (^a^, ^b^ or ^c^) are statistically different from each other (*p* < 0.05).

**Table 2:** Chemical structures and in vitro inhibitory activities of compounds **32**, **33**, **38**, **46**, and the positive control, **CP-91149**, against GPa activity, with 0 mM, 5 mM, and 10 mM of glucose concentrations.

	0 mM of Glucose	5 mM of Glucose	10 mM of Glucose
	GPa Inhibitory Activity (%) or IC_50_ Value		
** 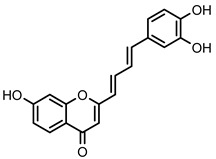 ** **2-Styrylchromone-related derivative 32**	**16.7 ± 1.5** ^(b,c)^	**13.4 ± 1.6** ^(b)^	**10.8 ± 1.2** ^(c)^
** 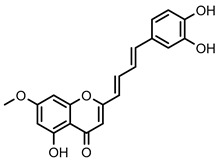 ** **2-Styrylchromone-related derivative 33**	**15.9 ± 1.1** ^(b,c)^	53.9 ± 2.5% ^40 μM^	<30% ^40 μM^
** 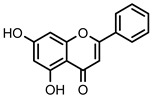 ** **Flavonoid 38** Chrysin	**19.3 ± 1.5** ^(b)^	**8.8 ± 0.8** ^(b)^	**5.2 ± 0.3** ^(b)^
** 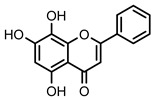 ** **Flavonoid 46** Norwogonin	**13.2 ± 1.4** ^(c)^	**8.9 ± 1.0** ^(b)^	**3.7 ± 0.5** ^(a; b)^
** 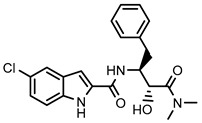 ** **Positive control** **CP-91149**	**0.58 ± 0.09** ^(a)^	**0.39 ± 0.05** ^(a)^	**0.22 ± 0.04** ^(a)^

Mean of the percent of inhibition ± SEM represented in at least three independent experiments. Concentrations shown in superscript indicate the highest concentrations that could be tested without interferences with the methodology. The IC_50_ values (represented in bold) with different lowercase superscript letters (^a^, ^b^ or ^c^) are statistically different from each other (*p* < 0.05).

## Data Availability

The data presented in this study are available on request from the corresponding author.

## Supplementary Materials

The following are available online at https://www.mdpi.com/article/10.3390/nu14020306/s1, Figure S1: Cross docking performance for the two best scoring methods selected from self-docking—LeDock and AutoDock; Figure S2: Interaction profile for Styrylpyrazoles; Figure S3: Interaction profile for 2-styrylchromones and 2-styrylchromone-related derivatives; Figure S4: Interaction profile for Flavonoids; Figure S5: Distribution of docking scores for the library of compounds developed in this work, shown with respect to inactive and active compounds (when applicable); Figure S6: Distribution of docking scores and ligand efficiency (LE) values for ChEMBL actives and inactives; Figure S7: X-ray contacts observed in the PDBs considered in cross-docking; Table S1: PDB structures used in this study; Table S2: Activities, docking scores and ligand efficiency (LE) across all compounds developed in this work.
