# Supplementary material for: An In Silico and an In Vitro Inhibition Analysis of Glycogen Phosphorylase by Flavonoids, Styrylchromones, and Pyrazoles
Source: Nutrients. 2022 Jan 12;14(2):306. doi: 10.3390/nu14020306 (PMC8781192; doi:10.3390/nu14020306)
Supplement: Supplementary file 1 [file nutrients-14-00306-s001.zip › nutrients-1529822-supplementary.pdf]

Supporting Information

Supporting Table S1. PDB structures used in this study.

PDB code	Resolution (Å)	Species
1L5Q	2.25	*Homo sapiens*
1L5R	2.1	
1L5S	2.1	
1L7X	2.3	
3DD1	2.57	
3DDS	1.8	
3DDW	1.9	
1Z8D	2.3	
1C8K	1.76	*Oryctolagus cuniculus*
1C8L	2.3	
1E1Y	2.23	
1GFZ	2.3	
1UZU	2.3	
1Z62	1.9	
2G9R	2.07	
2G9U	2.15	
3BCR	2.14	
3BD7	1.9	
3BDA	2	
3EBP	2	
4YUA	2	
4Z5X	2.1	
6Y55	2.38	
6Y5C	2.4	
6Y5O	2.33	
8GPB	2.2	
3EBO	1.9	

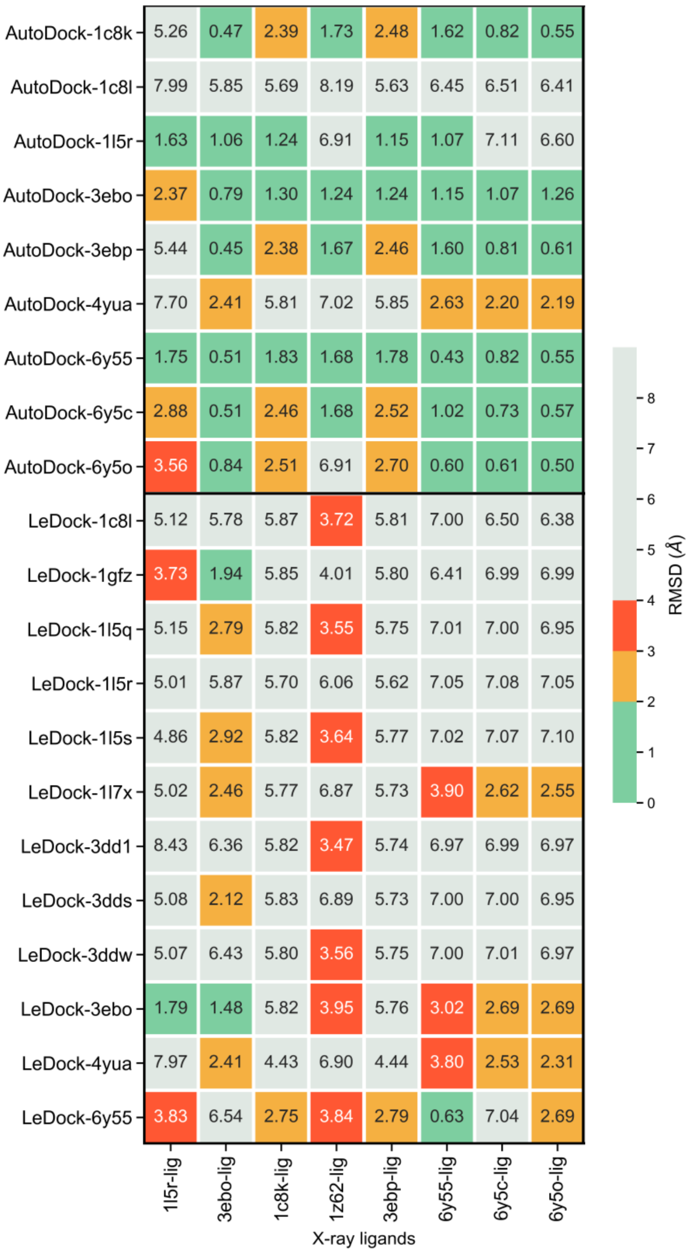

Supporting Figure S1. Cross docking performance for the two best scoring methods selected from self-docking - LeDock and AutoDock. This matrix resulted from docking each X-ray ligand onto every available protein PDB structure and through comparison of the top-scoring pose with the corresponding X-ray pose.

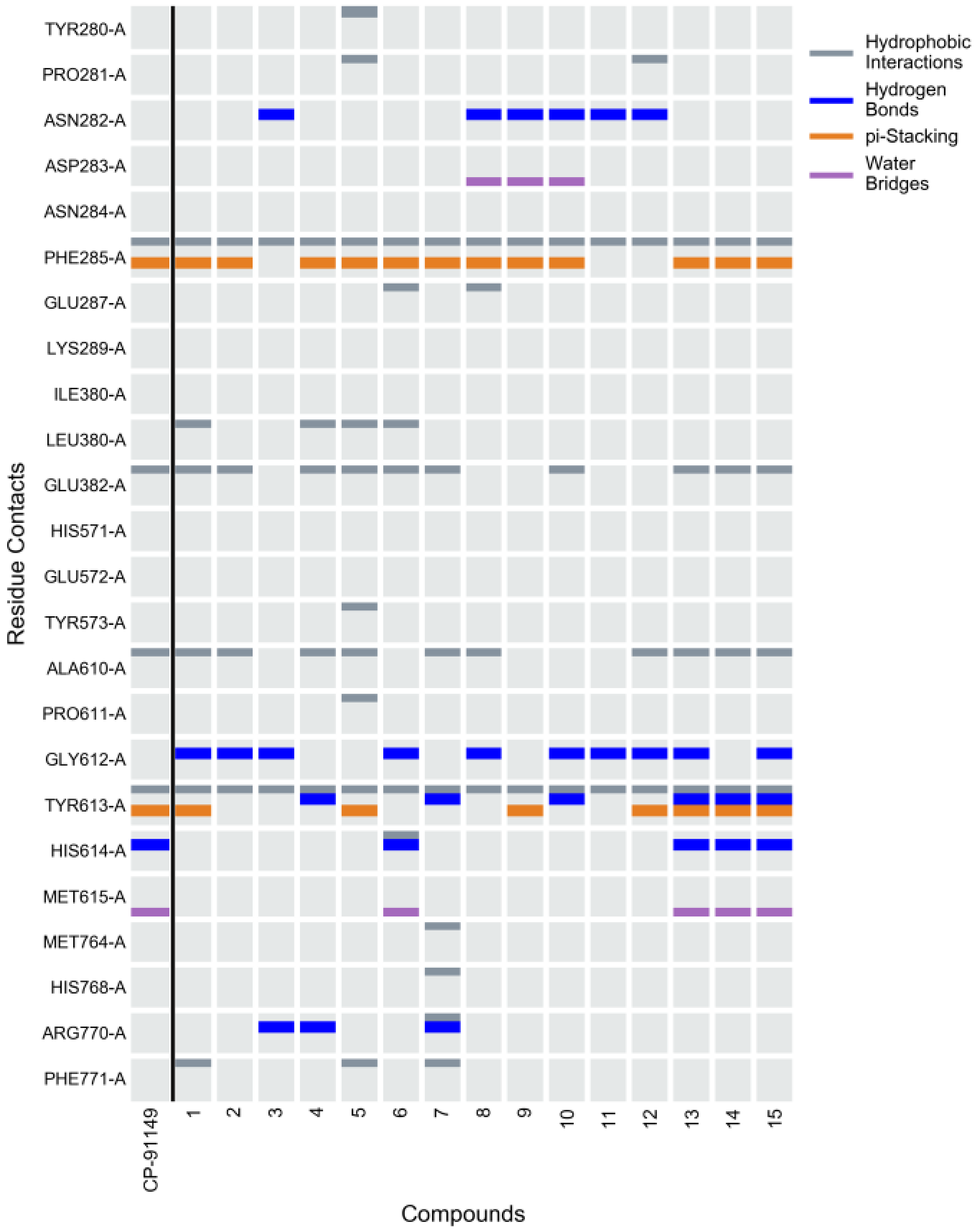

Supporting Figure S2. Interaction profile for Styrylpyrazoles. The X axis shows compounds sorted by activity and the black line separates actives from inactives.

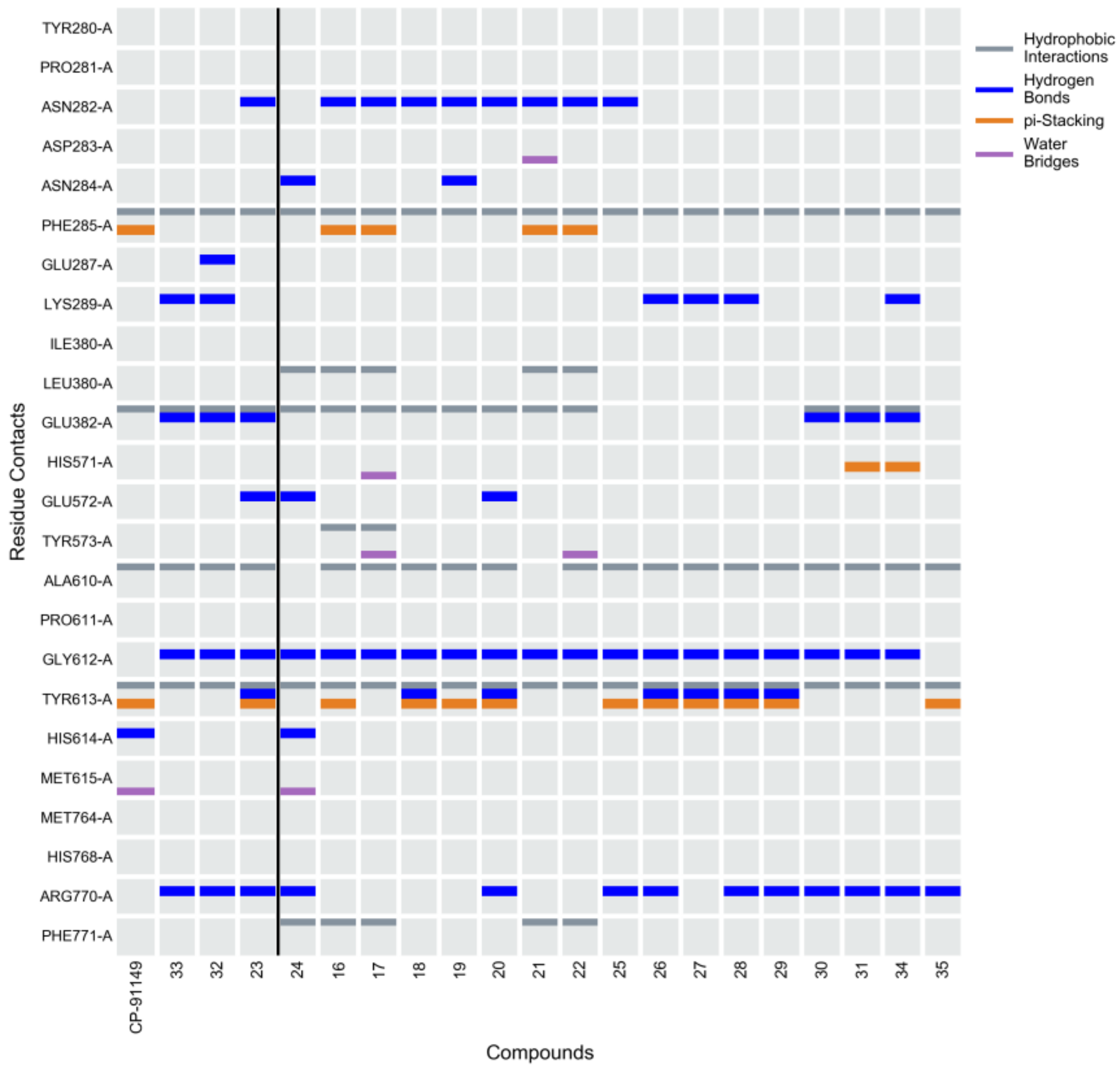

Supporting Figure S3. Interaction profile for 2-styrylchromones and 2-styrylchromone-related derivatives. The X axis shows compounds sorted by activity and the black line separates actives from inactives.

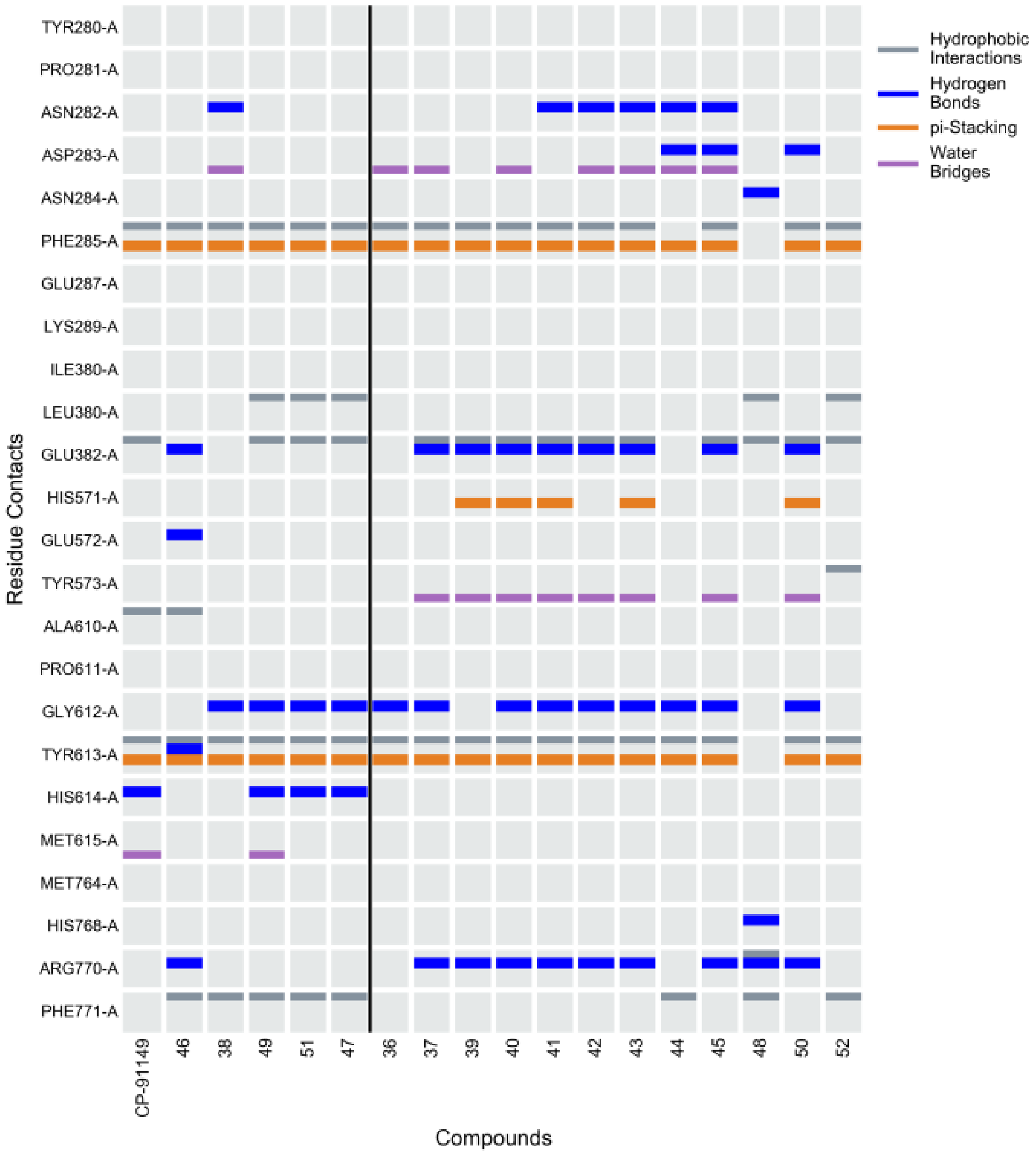

Supporting Figure S4. Interaction profile for Flavonoids. The X axis shows compounds sorted by activity and the black line separates actives from inactives.

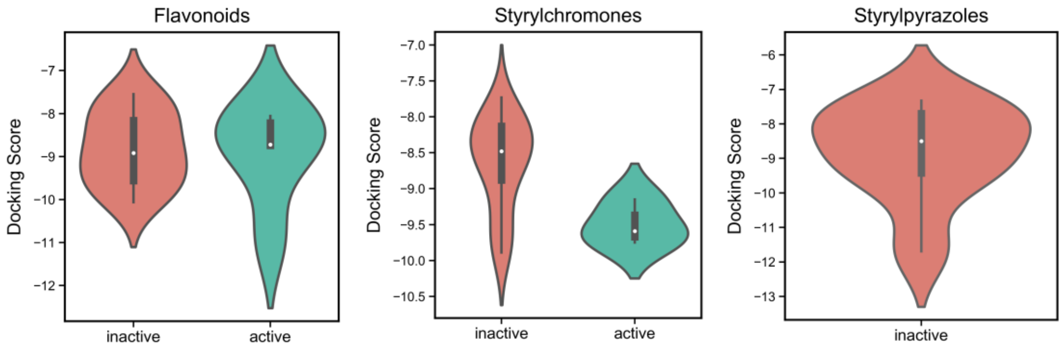

Supporting Figure S5. Distribution of docking scores for the library of compounds developed in this work, shown with respect to inactive and active compounds (when applicable).

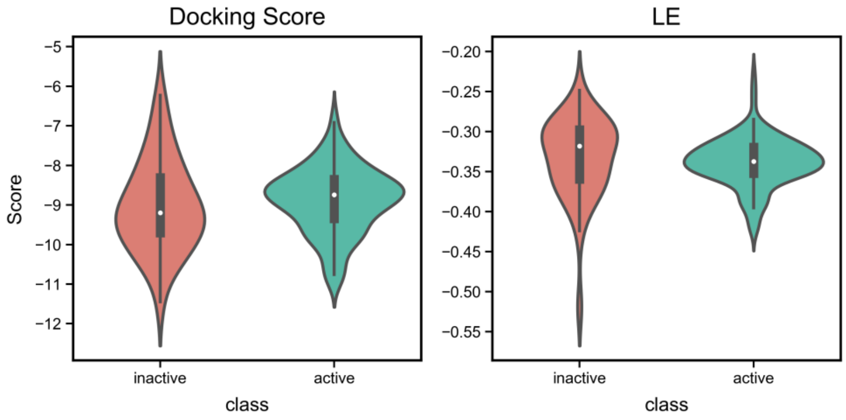

Supporting Figure S6. Distribution of docking scores and ligand efficiency (LE) values for ChEMBL actives and inactives.

Supporting Table S2. Activities, docking scores and ligand efficiency (LE) across all compounds developed in this work. Compounds are sorted inside their family, according to ascending docking score.

Family	Compound	Docking score	LE	IC_{50} (μM)
Flavonoids	49	-10.88	-0.286	20.5
	45	-10.06	-0.457	>50.0
	50	-9.87	-0.429	>50.0
	41	-9.58	-0.479	>50.0
	43	-9.55	-0.455	>50.0
	39	-9.11	-0.479	>50.0
	40	-9.0	-0.45	>50.0
	48	-8.84	-0.421	>50.0
	47	-8.74	-0.437	23.5
	46	-8.72	-0.436	13.2

	44	-8.41	-0.42	>50.0
	42	-8.28	-0.414	>50.0
	51	-8.22	-0.391	22.6
	38	-8.06	-0.424	19.3
	37	-7.82	-0.412	>50.0
	52	-7.75	-0.337	>50.0
	36	-7.55	-0.419	>50.0
2-Styrylchromones and 2-styrylchromone-related derivatives	31	-9.89	-0.412	>50.0
	24	-9.79	-0.408	>25.0
	33	-9.75	-0.375	15.9
	32	-9.59	-0.4	16.7
	30	-9.57	-0.416	>50.0
	34	-9.29	-0.372	50.0
	23	-9.15	-0.398	31.7
	20	-8.88	-0.404	>50.0
	25	-8.68	-0.347	>50.0
	21	-8.65	-0.412	>50.0
	19	-8.52	-0.387	>50.0
	35	-8.48	-0.314	>50.0
	29	-8.47	-0.339	>50.0
	26	-8.34	-0.309	>50.0
	18	-8.18	-0.39	>50.0
	22	-8.13	-0.37	>50.0
	28	-8.11	-0.324	>50.0
	16	-8.08	-0.404	>50.0
	27	-8.05	-0.298	>50.0
	17	-7.73	-0.368	>50.0
Styrylpyrazoles	14	-11.69	-0.292	>50.0
	15	-11.62	-0.277	>50.0
	13	-9.61	-0.3	>50.0
	7	-9.45	-0.286	>50.0
	12	-9.39	-0.335	>50.0
	6	-9.24	-0.289	>50.0
	5	-9.04	-0.301	>50.0
	11	-8.5	-0.405	>50.0
	10	-8.22	-0.411	>50.0
	2	-7.98	-0.38	>50.0
	1	-7.79	-0.39	>50.0
	3	-7.62	-0.346	>50.0
	4	-7.57	-0.315	>50.0
	8	-7.53	-0.327	>50.0
	9	-7.33	-0.282	>50.0
Control	CP-91149	-9.44	-0.337	0.58

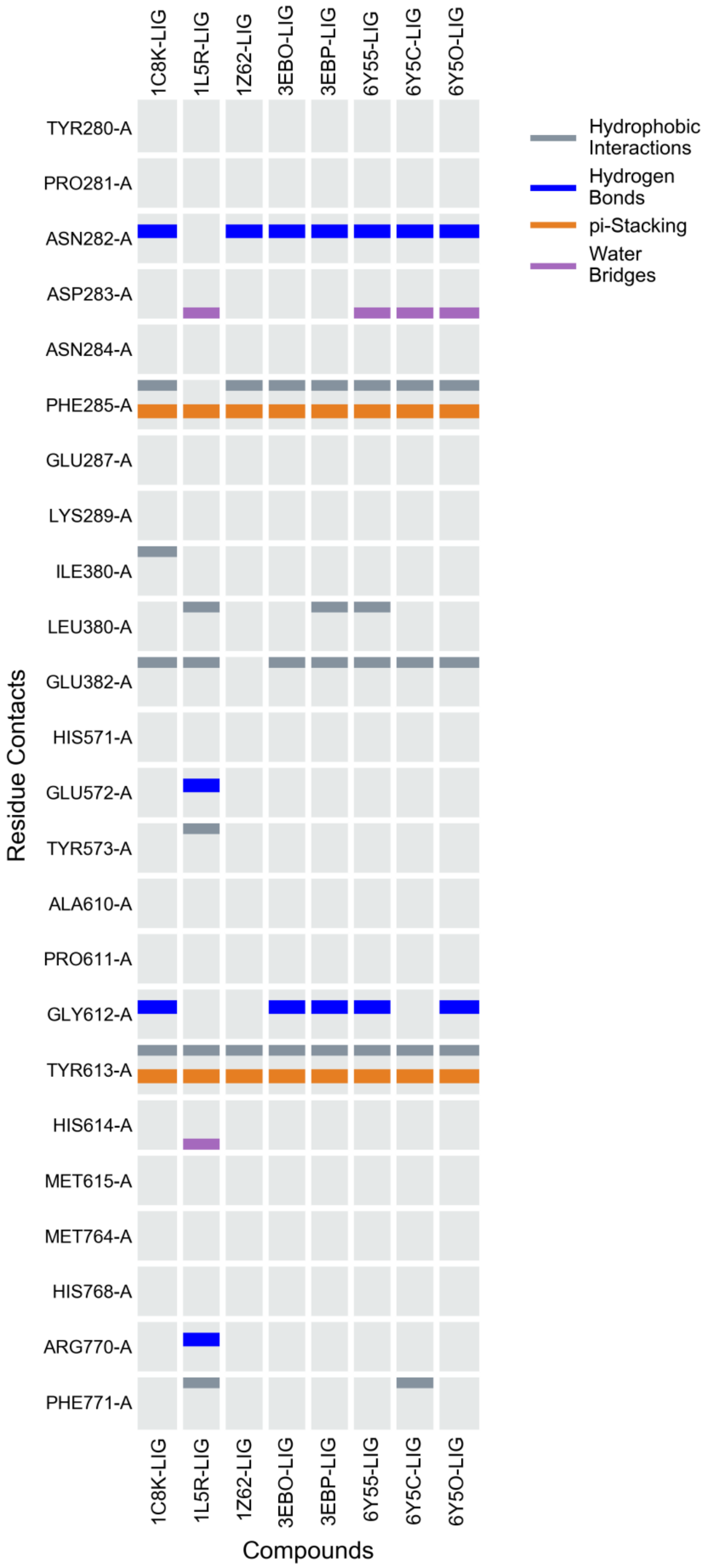

Supporting Figure S7. X-ray contacts observed in the PDBs considered in cross-docking.
